# Therapeutic effects of sesame oil on monosodium urate crystal-induced acute inflammatory response in rats

**DOI:** 10.1186/2193-1801-2-659

**Published:** 2013-12-07

**Authors:** Dur-Zong Hsu, Si-Jin Chen, Pei-Yi Chu, Ming-Yie Liu

**Affiliations:** Department of Environmental and Occupational Health, National Cheng Kung University Medical College, 138 Sheng-Li Road, Tainan, Taiwan; Sustainable Environment Research Centre, National Cheng Kung University, Tainan, Taiwan

**Keywords:** Gout, Hyperuricemia, Monosodium urate monohydrate, Mast cell, Inflammation, Arthritis

## Abstract

Sesame oil has been used in traditional Taiwanese medicine to relieve the inflammatory pain in people with joint inflammation, toothache, scrapes, and cuts. However, scientific evidence related to the effectiveness or action mechanism of sesame oil on relief of pain and inflammation has not been examined experimentally. Here, we investigated the therapeutic effect of sesame oil on monosodium urate monohydrate (MSU) crystal-induced acute inflammatory response in rats. Air pouch, a pseudosynovial cavity, was established by injecting 24 mL of filtered sterile air subcutaneously in the backs of the rats. At day 0, inflammation in air pouch was induced by injecting MSU crystal (5 mg/rat, suspended in sterilized phosphate buffered saline, pH 7.4), while sesame oil (0, 1, 2, or 4 mL/kg, orally) was given 6 h after MSU crystal injection. Parameters in lavage and skin tissue from the air pouches were assessed 6 h after sesame oil was given. Sesame oil decreased MSU crystal-induced total cell counts, tumor necrosis factor-α, interleukin (IL)-1β, and IL-6 levels in lavage and pouch tissue. Sesame oil significantly decreased leukocyte and neutrophil counts in lavage compared with MSU crystal alone group. Sesame oil decreased activated mast cell counts in skin tissue in MSU crystal-treated rats. Sesame oil significantly decreased nuclear factor (NF)-κB activity and IL-4 level in isolated mast cells from rats treated with MSU crystal. Furthermore, sesame oil decreased lavage complement proteins C3a and C5a levels in MSU crystal-treated rats. In conclusion, sesame oil shows a potent therapeutic effect against MSU crystal-induced acute inflammatory response in rats.

## Introduction

Sesame oil is derived from the plant species *Sesamum indicum* L., an herbaceous annual belonging to the Pedaliaceae family (Kubes & Grabger 1996). Constituents of sesame oil include olein, stearin, palmitin, myristin, linolein, sesamin, and sesamolin (Simon et al. 1984). In traditional Taiwanese medicine, sesame oil was used to relief the pain in people with joint pain, toothache, premenstrual syndrome, scrapes, and cuts. Recently, sesame oil has been proved to possess potent anti-inflammatory properties (Hsu & Liu 2002; Hsu & Liu 2004).

Gout, an extremely painful arthritis with relapsing inflammatory attacks, is a common inflammatory joint disease in men over the age of 40 (Turner et al. 1960; Terkeltaub 2006). The clinical symptoms of inflammatory responses are characterized by severe pain, oedema, and erythema in the joints (Pouliot et al. 1988). Nowadays, nonsteroidal anti-inflammatory drugs (NSAIDs) are the first-line drugs in managing acute attack of gout; however, their use is restricted in patients with renal inefficiency, gastrointestinal ulceration, and bleeding, or heart failure (Segasothy et al. 1995).

The formation of monosodium urate (MSU) crystals in the joint synovium under hyperuricemia condition is the main cause of acute gouty arthritis (Choi et al. 2005a). Neutrophils activation and infiltration to joint synovium after MSU crystal stimulation is a central feature of acute gouty inflammation (Choi et al. 2005b; Popa-Nita & Naccache 2010). Activated neutrophils produce a large number of proinflammatory cytokines, such as tumor necrosis factor (TNF)-α, interleukin (IL)-6, and IL-1β (Guerne et al. 1989; Terkeltaub et al. 1991; Choi et al. 2005c), all of which are involved in the pathogenesis and development of acute inflammation of gouty arthritis (Di Giovine et al. 1987).

Mast cell has become growing evident in the pathogenesis of diverse inflammatory diseases including arthritis (Krishnaswamy et al. 2001). Mast cell-related regulation is one of the important mechanisms underlying neutrophil activation (Schramm & Thorlacius 2004). Several chemotactic cytokines, such as IL-4 and IL-8, are produced from activated mast cells (Galli et al. 1991). Over-production of IL-4 by mast cell not only enhances the activation and infiltration of neutrophil (Kubes & Grabger 1996; Schramm et al. 2002; Park et al. 2007) but also increases the aggregation of mast cell in inflammatory site (Bischoff et al. 1999). However, the roles of mast cell in MSU crystal-induced acute gouty inflammation are still not clear. Furthermore, activation of complement pathways leads to the elaboration of C3a and C5a, which plays an important role in the degranulation of mast cell after MSU stimulation (Dalbeth & Haskard 2005). The aim of this study was to examine the therapeutic effect of sesame oil on MSU crystal-induced acute inflammation in rat air pouches.

## Materials and methods

### Chemicals

Uric acid, sesame oil, and mast cell stabilizer ketotifen were purchased from Sigma-Aldrich (St. Louis, MO).

### Animals

Male SPF Sprague–Dawley rats, weighing 200 to 250 g, were obtained from and housed in the Laboratory Animal Center of National Cheng Kung University. Rats were housed individually in a room with a 12-h light/dark cycle and central air conditioning (25°C, 70% humidity). Rats were allowed free access to tap water and were fed a rodent diet from Richmond Standard, PMI Feeds, Inc. (St. Louis, MO). The animal care and experimental protocols were in accordance with nationally approved guidelines. This study was reviewed and approved by the Laboratory Animal Center of National Cheng Kung University in Taiwan (NO. 102122).

### Rat air-pouch model

Subcutaneous air pouches were produced under ketamine anesthesia. Twenty-four milliliters sterile air was injected subcutaneously through a 0.25 μm microfilter into the backs of the animals to create a pseudosynovial cavity (Ferrari et al. 1996; Nagase et al. 1998). A second air injection was given 3 days after, if needed, to keep the air pouch inflated.

### MSU crystal preparation

Briefly, 8 g of uric acid was dissolved in 1.6 liter of boiling water containing 49 mL of 1 N NaOH. After adjusting the pH value to 7.4, the solution was cooled gradually at room temperature, and then stand overnight at 4°C. MSU crystals were recovered by centrifugation and dried by evaporative drying. Then, MSU crystals were sterilized by heating at 180°C for 2 h before experiments. After sonication, needle-shaped crystals (5–25 μm in length) were checked by using microscopy and then the MSU crystals were ready to used (Seegmiller et al. 1962). A Limulus amebocyte cell lysate assay was used to confirm the absence of endotoxin (less than 0.015 EU/ml) in the crystal preparation (Murakami et al. 2002).

### Collecting pouch lavage and counting leukocyte number in lavage

The air pouch lavage was collected by injecting 5 mL of PBS into the pouch then the lavage fluids were slowly instilled and withdrawn three times. Lavage was centrifuged (350 × *g* for 10 min, 4°C) and the cell pellet was resuspended with red blood cell lysing buffer (Sigma). After washing with PBS, viable cells were counted using trypan blue dye exclusion.

### Experimental design

#### Experiment I

Time course study of MSU crystal-induced acute inflammation in rat air pouches. Six rats were injected with MSU crystal (5 mg/rat, suspended in sterilized phosphate buffered saline, pH 7.4). The leukocyte infiltration was assessed 6 and 12 h after MSU injection.

#### Experiment II

The therapeutic effect of sesame oil on MSU crystal-induced acute inflammation. Rats were divided into five groups of six: Group I, negative control group; Group II, rats were injected MSU crystal (5 mg/rat) into air pouch; Group III-V, rats were given sesame oil 1, 2, and 4 mL/kg orally 6 h after MSU crystal, respectively. Pro-inflammatory mediators (TNF-α, IL-1β, and IL-6), leukocyte counts, neutrophil counts, and complement protein (C3a and C5a) levels were assessed in air pouch lavage 12 h after MSU crystal injection. In addition, mast cell counts in surrounding skin tissue were also assessed 12 h after MSU crystal injection.

#### Experiment III

Effects of sesame oil on nuclear factor (NF)-κB activation and IL-4 level in mast cells. Rats were divided into four groups of six: Group I, negative control group; Group II, rats were received sesame oil (4 mL/kg, orally) alone; Group III, rats were injected MSU crystal (5 mg/rat) into air pouch; and Group IV, rats were given sesame oil 6 h after MSU crystal injection. NF-κB activity and IL-4 levels in mast cells isolated from surrounding tissue were assessed 12 h after MSU crystal injection.

#### Experiment IV

Effect of mast cell stabilizer ketotifen on MSU crystal-induced acute inflammation. Rats were divided into five groups of six: Group I, negative control group; Group II, rats were injected MSU crystal (5 mg/rat) into air pouch; Group III-V, rats were given ketotifen orally (0.1, 1, and 10 mg/kg, respectively) 30 min before MSU crystal was given. A same dose of ketotifen was then given intravenously 15 min before MSU crystal. This dose regimen has been reported to avoid connective tissue mast cell degranulation (Kubes et al. 1993). Leukocyte numbers in pouch lavage were determined 12 h after MSU.

### Measuring TNF-α, IL-1β, IL-4, and IL-6 levels in lavage and surrounding skin tissue

TNF-α, IL-1β, IL-4, and IL-6 were measured using commercial enzyme-linked immunosorbent assay (ELISA) kits (DuoSet; R&D System, Minneapolis, MN). Briefly, a 96-well immunoassay plate was coated with 100 μL/well capture-antibody overnight at room temperature. After blocking step, lavage or skin tissue homogenate (tissue:water 1:10 w/v) was loaded into each well (100 μL/well) and were incubated at room temperature for 2 h. One hundred microliter of biotinylated rabbit anti-rat TNF-α, IL-1β, IL-4, and IL-6 antibodies were then added and incubated at room temperature for 2 h. After antibody capturing, streptavidin-conjugated horseradish peroxidase was added and was incubated at room temperature for 20 min. The peroxidase reaction was initiated by adding 100 μL of 3’,3’,5’,5’-tetramethylbenzidine/H_2_O_2_ (R&D systems) for 30 min, and then was stopped by adding 50 μL of 0.5 M H_2_SO_4_. The absorbance was measured at 450 nm using an enzyme-linked immunosorbent reader.

### Leukocyte counts in pouch lavage

The leukocyte count in the lavage fluid was determined manually using a hemocytometer. Erythrocytes are lysed in hypotonic buffer to avoid interfering determination of leukocyte count.

### Mast cell counts in inflammatory tissue

Briefly, skin tissue was fixed in 4% formaldehyde buffered with a phosphate solution (0.1 M [pH 7.4]) at room temperature. Tissue fragments were washed in phosphate buffer, dehydrated in graded concentrations of ethanol, and then embedded in paraffin. From each tissue, 4-μm-thin sections were cut, stained with toluidine blue, and examined under a light microscope.

### Mast cell isolation

The skin tissue was cut into 5-cm pieces and washed in Hanks' balanced salt solution (HBSS) containing 5% fetal bovine serum (FBS). To remove epithelial cells, the tissue was washed and stirred in HBSS containing ethylenediaminetetraacetic acid (EDTA). Then, skin tissue was cut into 2- to 5-mm pieces and enzymatically digested in HBSS (containing 20% FBS and 1 mg/mL of collagenase) for 90 min at 37°C. After digestion, the cells in supernatant were collected. Mast cells were purified by using Percoll (Pharmacia Fine Chemicals, Uppsala, Sweden). Isotonic Percoll solutions were prepared by dissolving nine parts of Percoll with one part of a ten-fold concentrated Hanks solution. An aliquot of 0.75 mL of the cell suspension in Hanks solution was added to 3.5 mL Percoll isotonic solution, resulting in a final density of 1.110 (Hachisuka et al. 1988). Then, 0.5 mL Hanks buffer was layered on top of the solution and centrifuged at 125 × *g* for 15 min. The uppermost 2.5 mL were removed. The remaining volume containing the mast cells was washed twice in Hanks solution. More than 90% of the isolated cells were mast cells (Enerbfick & Sevensson 1980).

### Assessing NF-κB activity in isolated mast cells

Nuclear protein extraction kit (Sigma) was used to isolate nuclear protein in skin tissue. NF-κB p65 was detected by the chemiluminescent NF-κB assay kits (Thermo Scientific, Inc, Rockford, USA). In brief, nuclear protein was loaded to the 96-well plate and bound to the biotin-Duplex. After incubation, the primary antibody and secondary antibody conjugated with HRP were added. Then chemiluminescent substrate was added and the luminescence was detected by using a luminescence analyzer (Fluoroskan Ascent FL, Thermo Fisher Scientific Inc, Waltham, MA) (Hsu et al. 2013).

### Complement system products C3a and C5a in pouch lavage

C3a and C5a levels in lavage were measured by using commercially ELISA kits (Uscn, USA). Briefly, standards or samples are then added to the microtiter plate wells coated with C3a or C5a biotin-conjugated polyclonal antibody. Then, avidin conjugated to horseradish peroxidase and TMB was added. The enzyme-substrate reaction is terminated by the addition of a sulphuric acid and the absorbance was measured spectrophotometrically at 450 nm.

### Statistical analysis

Data are means ± standard deviation (SD). Student’s *t*-test was used to make pairwise comparisons between the treatments in time course study. Group comparisons were done using one-way analysis of variance (ANOVA) and then Tukey’s honestly significant difference (HSD) post-hoc test. Significance was set at *P* < 0.05.

## Results and discussion

Sesame oil is derived from sesame seeds. The main constituents of sesame seed oil include fatty acids, and lignans. The fatty acids in sesame seed oil included palmitic acid (16:0; 7.0-12.0%), palmitoleic acid (16:1; less than 0.5%), stearic acid (18:0; 3.5-6.0%), oleic acid (18:1; 35-50%), linoleic acid (18:2; 35-50%), linolenic acid (18:3; less than 1%), and eicosenoic (20:1; less than 1%). The nonfat portion (1–2 wt%) of this oil contains ligans such as sesamin, sesamol, sesamolin, sesaminol, and episesamin. Sesame oil is unique due to its unusually high oxidative stability and anti-inflammatory property as compared to other edible oils (Kamal-Eldin et al. 1994). Sesame oil is rich with anti-oxidant, including sesamin, sesamolin, sesamol, and α-tocopherol. This strong antioxidant activity has been attributed mainly to the presence of α-tocopherol and antioxidative sesame lignans such as sesamin, sesamolin and sesamol (Chavali et al. 2001). Although sesame oil has been reported to protect many organ injuries models (Hsu et al. 2009; Hsu et al. 2011; Periasamy et al. 2013), it is the first study investigating the effect of sesame oil on gout model.

### Effect of MSU crystal on leukocyte infiltration in air pouch

**Figure 1:**
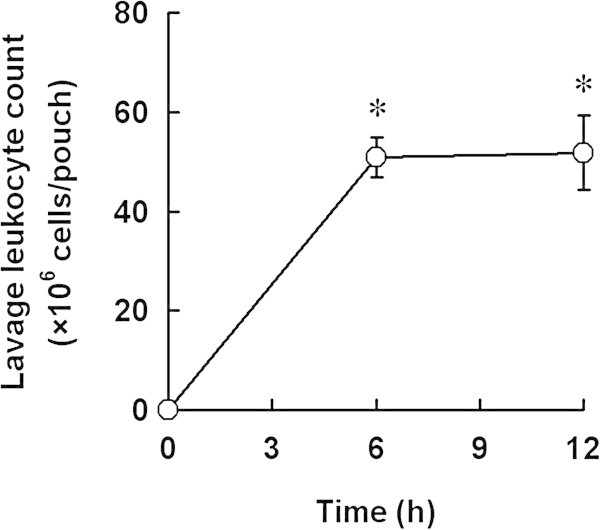
**Effect of MSU crystal on leukocyte infiltration in air pouch.** To establish the gout model, rats were injected with MSU crystal (5 mg/rat). The leukocyte infiltration was assessed 6 and 12 h after MSU injection. Data are means ± SD (n = 6). ^*^ *P* < 0.05 compared with 0 h.

To establish the air pouch gout model, time course of leukocyte infiltration induced by MSU crystal was conducted. MSU crystal significantly increased leukocyte number in air pouch at 6 and 12 h. However, there was no significant difference in leukocyte number between 6 and 12 h after MSU crystal treatment (Figure 1). To examine the therapeutic effect of sesame oil, in the following experiments, sesame oil was given to rats 6 h after MSU injection, and parameters were determined 6 h after sesame oil administration.

### Effects of sesame oil on MSU crystal-induced inflammatory response

Leukocytes infiltration plays a crucial role in the pathogenesis of gouty arthritis (Pouliot et al. 1988; Schiltz et al. 2002; So 2008). The formation of MSU in the joint synovium initiates inflammatory response in joint and leukocyte infiltration, which is the main characteristic of gouty arthritis (Popa-Nita et al. 2008). Among the infiltrated cells, neutrophil is regarded as a major source of pro-inflammatory mediators in MSU crystal-induced inflammatory response (Getting et al. 1997; Popa-Nita et al. 2008; Popa-Nita & Naccache 2010).

**Figure 2:**
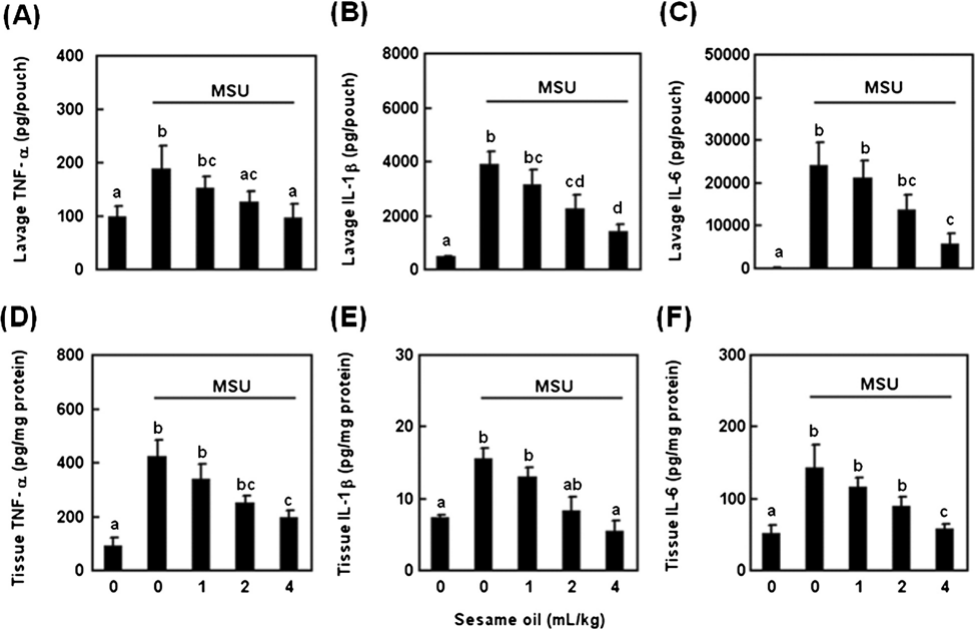
**Effect of sesame oil on MSU crystal-induced inflammatory mediator production in air pouch lavage and surrounding tissue.** To examine the therapeutic potential of sesame oil on gout, cytokines were detected. Rats were divided into five groups of six: Group I, negative control group; Group II, rats were treated with MSU crystal (5 mg/rat); Group III-V, rats were respectively given sesame oil 1, 2, and 4 mL/kg 6 h after MSU crystal. TNF-α, IL-1β, and IL-6 levels in both pouch lavage **(A-C)** and surrounding skin tissue **(D-F)** were measured 12 h after MSU crystal. Data are means ± SD (n = 6). Significant differences between groups were analyzed using one way ANOVA. Different letters above the bars indicate significant (*P* < 0.05) differences.

To examine the effect of sesame oil on inflammatory mediator production induced by MSU, TNF-α, IL-1β, and IL-6 levels were determined in pouch lavage as well as in surrounding skin tissue. MSU injection significantly increased both pouch lavage TNF-α (Figure 2A), IL-1β (Figure 2B), and IL-6 (Figure 2C) levles and skin tissue TNF-α (Figure 2D), IL-1β (Figure 2E), and IL-6 (Figure 2F) compared with that in negative control group. Sesame oil significantly decreased all tested pro-inflammatory cytokines production in a dose-dependent manner in both pouch lavage and skin tissue compared with that in MSU alone group (Figure 2A-F).

**Figure 3:**
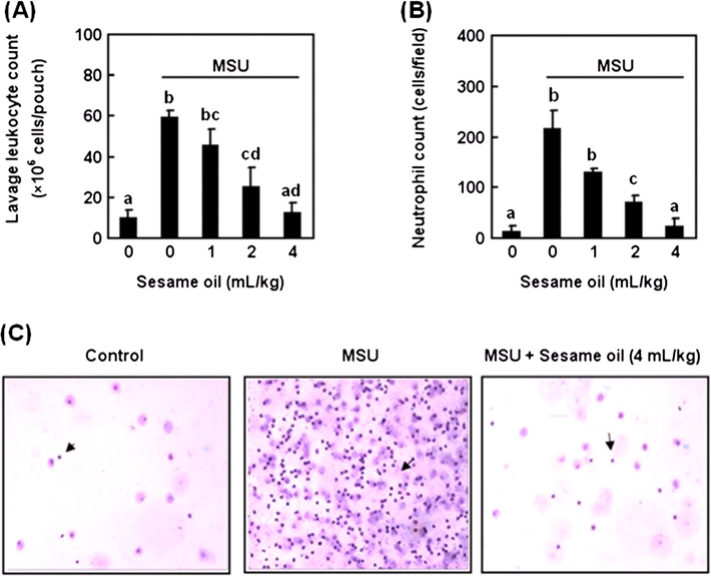
**Effects of sesame oil on MSU crystal-induced leukocyte and neutrophil infiltration in air pouch lavage.** To investigate the effect of sesame oil on gouty inflammation, leukocyte and neutrophil infiltration number were counted. Rats were divided into five groups of six: Group I, negative control group; Group II, rats were treated with MSU crystal (5 mg/rat); Group III-V, rats were respectively given sesame oil 1, 2, and 4 mL/kg 6 h after MSU crystal. Lavage leukocyte **(A)** and neutrophil **(B)** counts were assessed 12 h after MSU crystal. The arrows indicate neutrophil in pouch lavage slides (Hematoxylin and eosin stain; magnification = ×100) **(C)**. Data are means ± SD. Significant differences between groups were analyzed using one-way ANOVA. Different letters above the bars indicate significant (*P* < 0.05) differences.

To examine the therapeutic potential of sesame oil on MUS crystal-induced acute inflammation, the numbers of leukocyte and neutrophil in air pouch were determined. MSU increased leukocyte cell counts compared with that in negative control group. Sesame oil significantly decreased the leukocyte infiltration into air pouch in a dose-dependent manner compared with that in MSU alone group (Figure 3A). In addition, MSU crystal significantly increased neutrophil counts in pouch lavage, while sesame oil decreased neutrophil infiltration compared with that in MSU alone group (Figure 3B & C).

In summary, sesame oil significantly decreased neutrophil infiltration and pro-inflammatory cytokines production. It is likely that the inhibition of neutrophil activation is involved in sesame oil-exerted anti-inflammatory effect against MSU.

### Effect of sesame oil on mast cell accumulation and its degranulation in MSU crystal-induced acute inflammation

**Figure 4:**
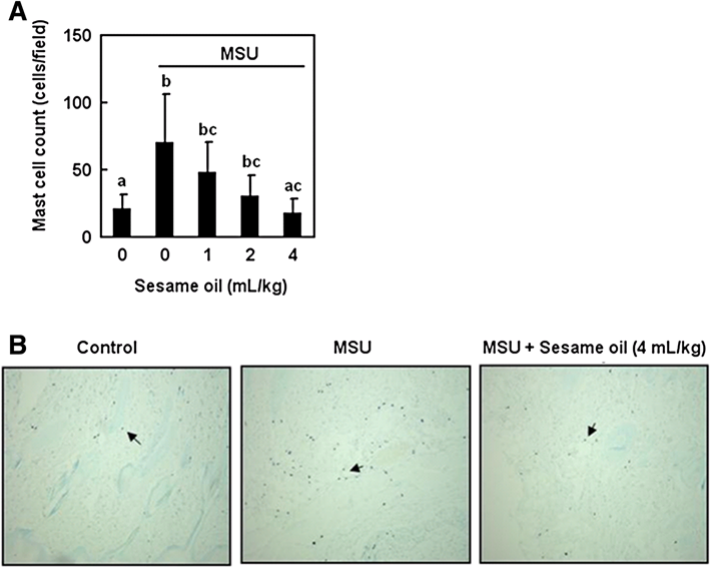
**Effect of sesame oil on mast cell accumulation in MSU crystal induced acute inflammation.** To investigate the role of mast cells on sesame oil-exerted anti-inflammatory effect on gout, active mast cell accumulation was assessed. Rats were divided into five groups of six: Group I, negative control group; Group II, rats were treated with MSU crystal (5 mg/rat); Group III-V, rats were respectively given sesame oil 1, 2, and 4 mL/kg 6 h after MSU crystal. Mast cell counts **(A)** in skin tissue were assessed 12 h after MSU crystal. Data are means ± SD (n = 6). Significant differences between groups were analyzed using one-way ANOVA. Different letters above the bars indicate significant (*P* < 0.05) differences. The arrows indicate activated mast cells **(B)**. (Toluidine blue stain; magnification = ×100).

Mast cell degranulation and aggravation are involved in the pathogenesis of gouty inflammation (Choi et al. 2005c). To examine the role of mast cell in sesame oil-exerted inhibitory effect in MSU crystal induced acute inflammation, mast cell counts in surrounding skin tissue were determined. MSU crystal significantly increased tissue mast cell counts compared with that in negative control group. Sesame oil significantly decreased mast cell counts compared with that in MSU alone group (Figure 4A & B).

**Figure 5:**
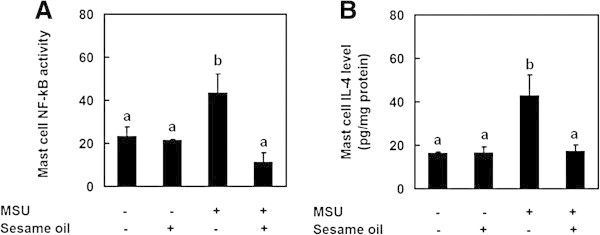
**Effects of sesame oil on NF-κB activity and IL-4 level in isolated mast cell in MSU crystal-treated rats.** To investigate mast cell degranulation in sesame oil-treated gout, NF-κB activity **(A)** and IL-4 level **(B)** were assessed. Rats were divided into four groups of six: Group I, negative control group; Group II, rats were received sesame oil (4 mL/kg, orally) alone; Group III, rats were injected MSU crystal (5 mg/rat) into air pouch; and Group IV, rats were given sesame oil 6 h after MSU crystal injection. NF-κB activity and IL-4 level in mast cells isolated from surrounding tissue were assessed 12 h after MSU crystal injection. Data are means ± SD (n = 6). Significant differences between groups were analyzed using one-way ANOVA. Different letters above the bars indicate significant (*P* < 0.05) differences.

Activation of mast cell leads to phosphorylation of tyrosine kinase and mobilization of internal Ca^2+^, followed by the activations of protein kinase C, mitogen-activated protein kinase (MAPKs), and NF-κB (Min et al. 2007). In addition, IL-4 may play an important role in MSU crystal-induced acute gouty inflammation. After activated, mast cell produces several chemotactic cytokines, including IL-4 (Popa-Nita & Naccache 2010). IL-4 enhances mast cell itself degranulation and accumulation in inflammatory area (Toru et al. 1998; Bischoff et al. 1999). Inhibiting IL-4 production decreases further mast cell degranulation and accumulation (Toru et al. 1998; Bischoff et al. 1999). Therefore, to examine the mechanism of sesame oil’s inhibitory effect on mast cell degranulation induced by MSU crystal, NF-κB activity and IL-4 level in isolated mast cell were assessed. MSU crystal increased NF-κB activity (Figure 5A) and IL-4 (Figure 5B) compared with negative control group, while sesame oil significantly decreased them compared that in MSU alone group. In addition, sesame oil alone did not alter NF-κB activity and IL-4 level compared with negative control group (Figure 5A & B).

**Figure 6:**
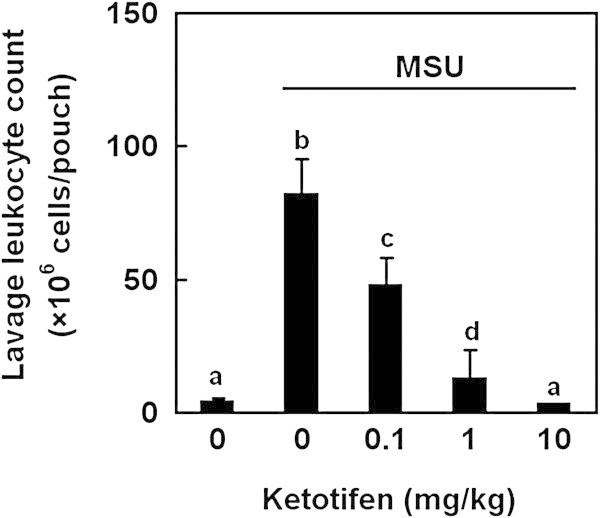
**Effects of mast cell stabilization on leukocyte counts in MSU crystal-induced acute inflammation.** To confirm the role of mast cell in gout, mast cell stabilizer ketotifen was examined in gout model. Rats were divided into five groups of six: Group I, negative control group; Group II, rats were injected MSU crystal (5 mg/rat) into air pouch; Group III-V, rats were given ketotifen 0.1, 1, and 10 mg/kg orally 30 min before MSU crystal. The same dose of ketotifen was then given intravenously as a bolus 15 min before MSU crystal. Leukocyte numbers in pouch lavage were determined 12 h after MSU. Data are means ± SD (n = 6). Significant differences between groups were analyzed using one-way ANOVA. Different letters above the bars indicate significant (*P* < 0.05) differences.

To confirm the role of mast cell in sesame oil-exerted anti-inflammatory effect against MSU-crystal, leukocyte counts in pouch lavage was determined in mast cell stabilizer ketotifen-treated rats. Ketotifen significantly decreased MSU-induced lavage leukocyte counts in a dose-dependent manner (Figure 6).

According to these results, sesame oil decreased the MSU crystal-induced activation of nuclear NF-κB in mast cell, and mast cell stabilizer ketotifen blockaded inflammatory cells infiltration in MSU crystal-treated rats. We suggest that inhibiting mast cell degranulation accounts for sesame oil’s anti-inflammatory effect in MSU crystal-induced gouty inflammation. Further, in the present study, sesame oil inhibited IL-4 release, mast cell degranulation and accumulation in inflammatory site. Therefore, we suggest that inhibiting IL-4 production in mast cell is involved in sesame oil-associated anti-inflammatory effect against MSU crystal challenge.

### Effects of sesame oil on complement proteins in MSU crystal-induced acute inflammation

Complement system activation is one of the major mechanisms in regulating mast cell degranulation (Erdei et al. 2004). Complement-derived peptides C3a and C5a have been reported to play an important role in the activation of mast cell in clinical studies (Hartmann et al. 1997). Activity of complement system is usually low in normal synovial fluid; however, it is greatly increased in the patients with acute gout (Pekin & Zvaifler 1964). In addition, MSU crystals can directly activate the classical pathway in the absence of immunoglobulin (Giclas et al. 1979).

**Figure 7:**
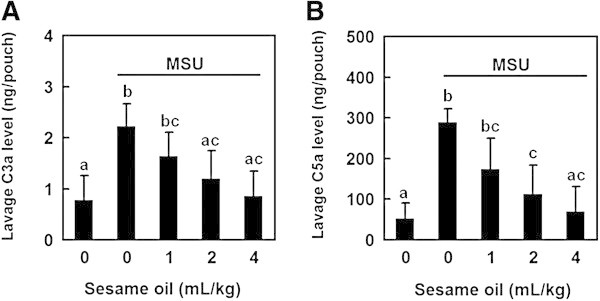
**Effect of sesame oil on complement system in MSU crystal-induced acute inflammation.** To examine the mechanism of sesame oil-exerted anti-inflammatory effect in gout, complement system was investigated. Rats were divided into five groups of six: Group I, negative control group; Group II, rats were treated with MSU crystal (5 mg/rat); Group III-V, rats were given sesame oil 1, 2, and 4 mL/kg, respectively, 6 h after MSU crystal. Lavage C3a **(A)** and C5a **(B)** levels were assessed 12 h after MSU crystal was given. Data are means ± SD. Significant differences between groups were analyzed using one-way ANOVA. Different letters above the bars indicate significant (*P* < 0.05) differences.

To examine the mechanism of sesame oil-exerted inhibitory effect on MSU crystal-induced acute inflammation, complement system products C3a and C5a levels in pouch lavage were determined. MSU crystal increased C3a and C5a production in pouch lavage compared with negative control group. Sesame oil at the doses of 2 and 4 mL/kg significantly decreased C3a and C5a production compared with MSU alone group (Figure 7A and B). It is likely that sesame oil decreases the direct stimulation of MSU crystal on complement system. We suggest that inactivating complement system is involved in sesame oil-associated inhibition of mast cell degranulation in MSU crystal-induced acute gouty inflammation, at least partially.

Sesamol (3, 4-Methylenedioxyphenol) (C_7_H_6_O_3_), one of the sesame seed oil lignans, has been generally regarded as the main anti-oxidative and anti-inflammatory component in sesame seeds. Sesamol is reported to decrease the inflammatory response and attenuates the associated organ damage in septic rats (Hsu et al. 2006; Chu et al. 2010). Administration of sesamol downregulates the LPS-induced production of TNF-α, IL-1β, and nitric oxide in serum, and expression of inducible nitric oxide syhthase (iNOS) in leukocytes, and because of the anti-inflammatory effect, multiple organ injury and mortality can be decreased. Further, sesamol inhibits the activation of macrophages. Sesamol affects macrophages to downregulate pro-inflammatory mediators production, including TNF-α, IL-1β, and NO, as well as NF-κB activation (Chu et al. 2009). It is possible that sesamol is the main effective factor in sesame oil-exerted anti-inflammatory effect on gout. However, the effects of sesamol on gouty inflammation and on mast cell activations have never been investigated. The effect of sesamol on gout needs further investigations.

## Conclusion

In conclusion, sesame oil showed a potent therapeutic effect against MSU-induced inflammation through inhibiting mast cell activation in MSU crystal-stimulated air pouch. Furthermore, sesame oil may have the potential in treating patients with gouty arthritis in the future.

## Abbreviations

NSAIDs: Nonsteroidal anti-inflammatory drugs
MSU: Monosodium urate
TNF: Tumor necrosis factor
IL: Interleukin
NF: Nuclear factor.
